# ZnO-Layered Double Hydroxide@Graphitic Carbon Nitride Composite for Consecutive Adsorption and Photodegradation of Dyes under UV and Visible Lights

**DOI:** 10.3390/ma9110927

**Published:** 2016-11-15

**Authors:** Luhong Zhang, Li Li, Xiaoming Sun, Peng Liu, Dongfang Yang, Xiusong Zhao

**Affiliations:** 1 School of Chemical Engineering, The University of Queensland, Brisbane, QLD 4072, Australia; l.zhang9@uq.edu.au (L.Z.); x.sun5@uq.edu.au (X.S.); p.liu2@uq.edu.au (P.L.); d.yang@uq.edu.au (D.Y.); 2 Australian Institute for Bioengineering and Nanotechnology, The University of Queensland, Brisbane, QLD 4072, Australia; l.li2@uq.edu.au

**Keywords:** zinc oxide, layered double hydroxide, graphitic carbon nitride, adsorption, photocatalysis

## Abstract

In this work, a ZnO-layered double hydroxide@graphitic carbon nitride composite (ZnO-LDH@C_3_N_4_) was synthesized via co-precipitation method with solvothermal treatment. The structure and morphology of ZnO-LDH@C_3_N_4_ composite were characterized using X-ray diffraction (XRD), Fourier transform infrared spectroscopy (FT-IR), scanning electron microscopes/transmission electron microscopes (SEM/TEM), N_2_ adsorption/desorption, ultraviolet visible diffuse reflectance spectroscopy (UV-Vis-DRS), photoluminescence spectrometer (PL) and electrochemical impedance spectroscopy (EIS). The adsorption and photocatalytic properties of ZnO-LDH@C_3_N_4_ composite towards the organic dyes: Orange II sodium salt (OrgII, an anionic azo dye) and methylene blue (MB, a cationic azo dye) were investigated. Compared to ZnO-LDH and g-C_3_N_4_, the ZnO-LDH@C_3_N_4_ composite displayed an excellent performance in both adsorption and photocatalytic degradation of the organic dyes. Moreover, a combination of ZnO-LDH and g-C_3_N_4_ significantly improved the photocatalytic performance of ZnO-LDH and g-C_3_N_4_ under visible-light irradiation. The adsorption and photocatalytic mechanism were also investigated.

## 1. Introduction

Several commonly used dyes that are toxic and mutagenic for aquatic organisms can even be carcinogenic for humans [1]. Adsorption and photodegradation are the common methods that can be used to eliminate the dyes in water. Recently, layered double hydroxides (LDHs), a family of anionic clay, have attracted much attention in the removal of organic dyes from wastewater due to their unique properties such as high ionic exchange capacity, tunable particle size, large surface area and various composition as well as good stability [2,3,4]. LDHs and the layered double oxide (LDO) by thermal treatment of LDH have displayed high adsorption capacity for organic dyes such as Orange II (Org II) [5,6,7]. Removal of the adsorbed organic dyes in the solid adsorbent is an essential step if the technology is to be used in practice. Photocatalytic degradation is a sustainable and green approach to removing the adsorbed dyes. However, these LDHs and their derivatives are only photocatalytically active under UV light [6]. In addition, these LDH-based materials showed little adsorption towards cationic dyes and did not show good photocatalytic performance under visible lights.

Graphitic carbon nitride (g-C_3_N_4_), a polymeric material with an electron-rich property, has been shown to be a promising visible light photocatalyst due to its suitable bandgap (2.7 eV), good stability, ease of preparation in addition to the feature of being environmentally friendly [8,9,10,11]. However, g-C_3_N_4_ is limited by its low quantum efficiency and high recombination rate of excited charges [8,12]. To improve its photocatalytic properties, g-C_3_N_4_ has been combined with inorganic semiconductor materials to develop composite photocatalysts with improved properties [13,14,15]. Di et al. reported that a sphere-like g-C_3_N_4_/BiOI composite exhibited a higher photocatalytic activity in the photodegradation of dyes than pure BiOI, which was due to the enhanced electron-hole separation and broadened light absorption range [16]. Jiang et al. [17] and Zhou et al. [18] both reported the heterojunction between g-C_3_N_4_ and TiO_2_, which showed effects on water pollution treatment, hydrogen production and an efficient photoreduction of CO_2_ to CO, respectively. Song and coworkers synthesized a g-C_3_N_4_(CN)-sensitized NaNbO_3_(NN) substrated II-type heterojunction, which not only exhibited narrower bandgap compared with NN but also displayed excellent photocatalytic activity for dyes and tetracycline degradation under visible-light irradiation [19]. Lan et al. reported that the Zn-In mixed metal oxide/g-C_3_N_4_ (ZnIn-MMO/g-C_3_N_4_) nanohybrids showed stronger absorption in the visible region than the pristine ZnIn-MMO, by exhibiting enhanced photodegradation activity for Rhodamine B under visible-light irradiation in comparison with pure g-C_3_N_4_ and ZnIn-MMO [12]. In addition, inter-electron transfer between g-C_3_N_4_ and ZnO heterogeneous junction was also reported and realized for photocatalytic degradation of pollutants [20,21,22,23]. Thus, the introduction of g-C_3_N_4_ to ZnO-LDH composite may tune the bandgap of ZnO-LDH composite and facilitate the separation of excited charges to improve the photocatalytic performance.

In this paper, we report a simple method for the design and preparation of a ZnO-LDH@C_3_N_4_ composite with improved adsorption and photocatalytic properties towards both anionic and cationic dyes present in water.

## 2. Experimental Details

### 2.1. Materials

All the chemicals used in this work such as urea, Zn(NO_3_)_2_∙6H_2_O, Al(NO_3_)_3_∙9H_2_O, NaOH, ZnO, methylene blue (C_16_H_18_ClN_3_S) and Orange II sodium salt (C_16_H_11_N_2_NaO_4_S) were purchased from Sigma-Aldrich with analytical grade and used without further purification. Milli-Q water (ultrapure laboratory grade water) was utilized in all experiments.

### 2.2. Preparation of ZnO-LDH@C_3_N_4_ Composite

#### 2.2.1. Synthesis of g-C_3_N_4_ Nanosheets

The g-C_3_N_4_ used in this study was prepared by calcining urea at 550 °C for 3 h in an alumina crucible according to the literature [24]. In brief, a given amount of urea was dried at 80 °C for 24 h, then calcined at 550 °C for 3 h. The heating rate of calcination was 2 °C/min. After cooling, the yellowish powder was collected and washed with 10% HNO_3_ solution and Milli-Q water three times by centrifugation.

#### 2.2.2. Preparation of ZnO-LDH@C_3_N_4_ Composite

**Scheme 1:**
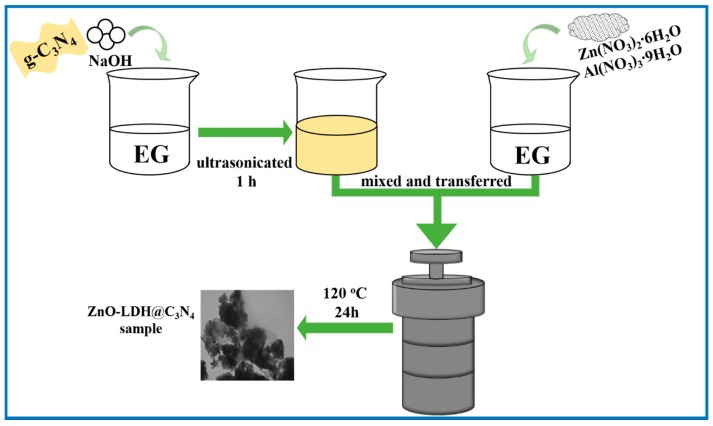
Schematic representation of the synthesis process of the ZnO-LDH@C_3_N_4_ composite.

The preparation of ZnO-LDH@C_3_N_4_ composite is illustrated in Scheme 1. First, 0.42 g of g-C_3_N_4_ and 2.80 g of NaOH were added to 40 mL of ethylene glycol (EG) under stirring. Second, 5.95 g of Zn(NO_3_)_2_∙6H_2_O and 3.75 g of Al(NO_3_)_3_∙9H_2_O (with a molar ratio of Zn/Al = 2:1) were added to another 40 mL of EG. Then, these two EG suspensions were combined under stirring. After 0.5 h, the suspension was transferred to a 100 mL Teflon-lined stainless steel autoclave for solvothermal treatment at 120 °C for 24 h. Finally, the solids were collected and washed with ethanol two times and Milli-Q water three times by centrifugation and dried in an oven at 80 °C for 24 h. The yield of the solids was 2.88 g.

#### 2.2.3. Synthesis of ZnO-LDH

A ZnO-LDH sample was prepared via a solvothermal method with the same parameters as preparation of the ZnO-LDH@C_3_N_4_ composite without adding g-C_3_N_4_.

### 2.3. Characterization

X-ray diffraction (XRD) patterns were collected on a Shimadzu diffractometer (XRD–6000, Tokyo, Japan) using the reflection mode with Cu Kα radiation at a scanning rate of 2°/min with 2θ ranging from 5° to 80°. X-ray photoelectron spectroscopy (XPS, Kratos Analytical Ltd., Manchester, UK) measurements were performed on a Kratos Axis ULTRA X-ray photoelectron spectrometer with a 165 mm hemispherical electron energy analyzer and monochromatic Al K*α* X-ray source (1486.6 eV) at 225 W (15 kV, 15 mA) with a charge neutralizer. The binding energies were calibrated using the C 1s peak at 284.6 eV. Fourier transform infrared spectroscopy (FT-IR, SHIMADZU, Kyoto, Japan) of the samples were recorded on a Shimadzu IRAffinity-1 spectrophotometer. The morphologies and size of the samples were characterized by using scanning electron microscope (SEM, JEOL JSM-6460LA, JEOL Ltd., Tokyo, Japan), transmission electron microscope (TEM, JEOL JEM-1010, JEOL Ltd., Tokyo, Japan) and high-resolution transmission electron microscope (HRTEM, JEOL JEM-2100, JEOL Ltd., Tokyo, Japan). The elemental analysis and mapping were done using energy dispersive X-ray spectroscopy (EDX). Light Scattering Electrophoresis (LSE, Malvern Instruments Malvern, Worcestershire, UK) in Nanosizer Nano ZS was used to analyze the zeta potentials of g-C_3_N_4_, ZnO-LDH, ZnO-LDH@C_3_N_4_ suspensions. The specific surface areas of the samples were calculated using the Brunauer-Emmette-Teller (BET, Micromeritics Instrument Corporation, Norcross, GA, USA) method based on the nitrogen adsorption isotherms measured on a Micromeritics TriStar II 3020 at liquid-nitrogen temperature. Thermogravimetric- differential thermal analysis (TG-DTA, Shimadzu DTG-60A analyser, SHIMADZU, Kyoto, Japan) was conducted in air from room temperature to 700 °C at a heating rate of 10 °C min^−1^. Diffuse reflectance spectra (DRS) were recorded on a Shimadzu UV-2600 spectrometer equipped with an integrating sphere ISR-3100 using BaSO_4_ as the reference. Photoluminescence (PL) spectra were measured on a Fluorescence Spectrometer (FLS 920, Edinburgh Instruments, Edinburgh, UK). Electrochemical impedance spectroscopy (EIS) measured in a 6 M KOH solution with sinusoidal ac perturbation of 5 mV over a frequency range from 0.1 to 1 × 10^6^ Hz.

### 2.4. Adsorption Measurements

The adsorption of the dye pollutants was measured using a batch mode. For the adsorption of OrgII over the solid samples (including ZnO-LDH, ZnO-LDH@C_3_N_4_, and C_3_N_4_), 5 mg of a solid sample was added to 100 mL of an OrgII solution (the concentration was 50 mg/L) in the dark under stirring for 24 h to achieve sorption equilibrium. For the adsorption of MB, 100 mg of a solid sample was added to 100 mL MB solution (the concentration was 10 mg/L) in the dark under stirring for 1 h to achieve the adsorption equilibrium. After a given time interval, 5 mL of the aliquot was taken and filtrated through a membrane (0.45 µm). The OrgII and MB concentrations were analyzed using a Shimadzu UV-2600 UV-Vis spectrophotometer.

### 2.5. Photocatalytic Degradation Test of Cationic Dye Methylene Blue (MB)

After achieving the adsorption equilibrium, the photocatalytic degradations of MB on the adsorbents were undertaken in an open thermostatic photoreactor under UV light and visible light, respectively. The UV-vis light resource was obtained from a 200 W mercury lamp. Visible-light irradiation was operated via adding a 450 nm cut-off filter on the mercury lamp. At given time intervals during irradiation, 5 mL of aliquots were extracted using a syringe and filtered through a membrane (0.45 µm). The concentration of MB in the solutions was analyzed using a Shimadzu UV-2600 UV-Vis spectrophotometer.

### 2.6. Intermediate Species of Photocatalytic Degradation

The species generated in the photocatalytic system were analyzed using tertbutyl alcohol (*t*-BuOH) and ethylenediaminetetraacetic acid disodium salt (EDTA-2Na). In detail, *t*-BuOH or EDTA-2Na (1 mmol) was mixed with the MB solutions before adding ZnO-LDH@C_3_N_4_ composite. Then the photocatalytic degradation of MB was performed in the thermostatic photoreactor under UV or visible-light irradiation with a similar process. The active species generated in the photocatalytic process could be detected through trapping by *t*-BuOH and EDTA-2Na [23].

## 3. Results and Discussion

### 3.1. Characterization of Samples

**Figure 1:**
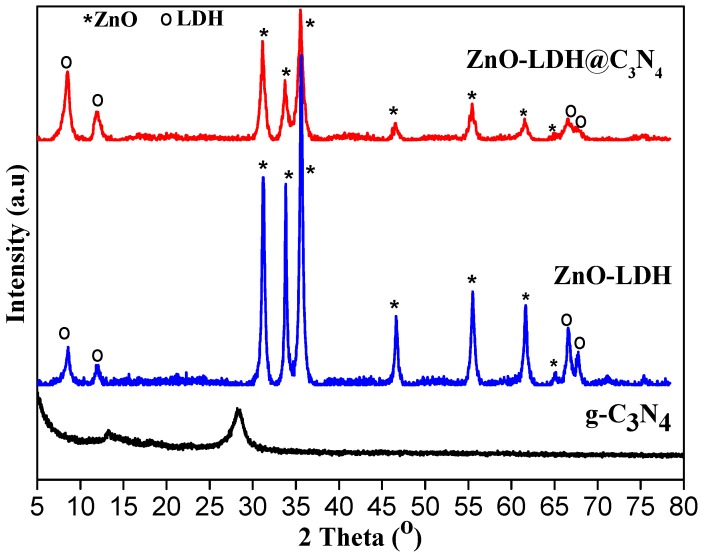
XRD patterns of g-C_3_N_4_, ZnO-LDH and ZnO-LDH@C_3_N_4_.

The XRD patterns of g-C_3_N_4_, ZnO-LDH and ZnO-LDH@C_3_N_4_ are shown in Figure 1. Two diffraction peaks at 13.4° and 28.4° two theta can be seen from the g-C_3_N_4_ sample, attributing to the diffractions of the (100) and (002) planes of g-C_3_N_4_. The (100) peak of g-C_3_N_4_ represents the heptazine unit with an interplanar separation of 0.66 nm and (002) peak represents the graphitic-like layer with an interlayer distance of 0.31 nm [25]. The XRD pattern of ZnO-LDH showed the presence of both ZnAl-LDH and ZnO [6]. The strong peaks at 31.2°, 33.8°, and 35.6° two theta found in ZnO-LDH sample are indexed to the diffraction of the (100), (002) and (101) planes of ZnO (JCPDS No. 05-0664), indicating the formation of ZnO in the sample [26,27]. The diffraction peaks at 8.6° and 12.1° are ascribed to the diffractions of the (003) and (006) planes of ZnAl-LDH in the ZnO-LDH sample. The interlayer distance of ZnAl-LDH in the ZnO-LDH sample d_(003)_ was 1.03 nm, which is larger than that of conventional carbonate intercalated LDH (0.73 nm), resulting from the intercalation of EG during solvothermal treatment [28,29,30]. The ZnO-LDH@C_3_N_4_ sample exhibited an XRD pattern similar to that of the ZnO-LDH sample. No characteristic diffraction peaks of g-C_3_N_4_ can be seen, probably due to the relatively weak intensity of the XRD peaks of g-C_3_N_4_.

**Figure 2:**
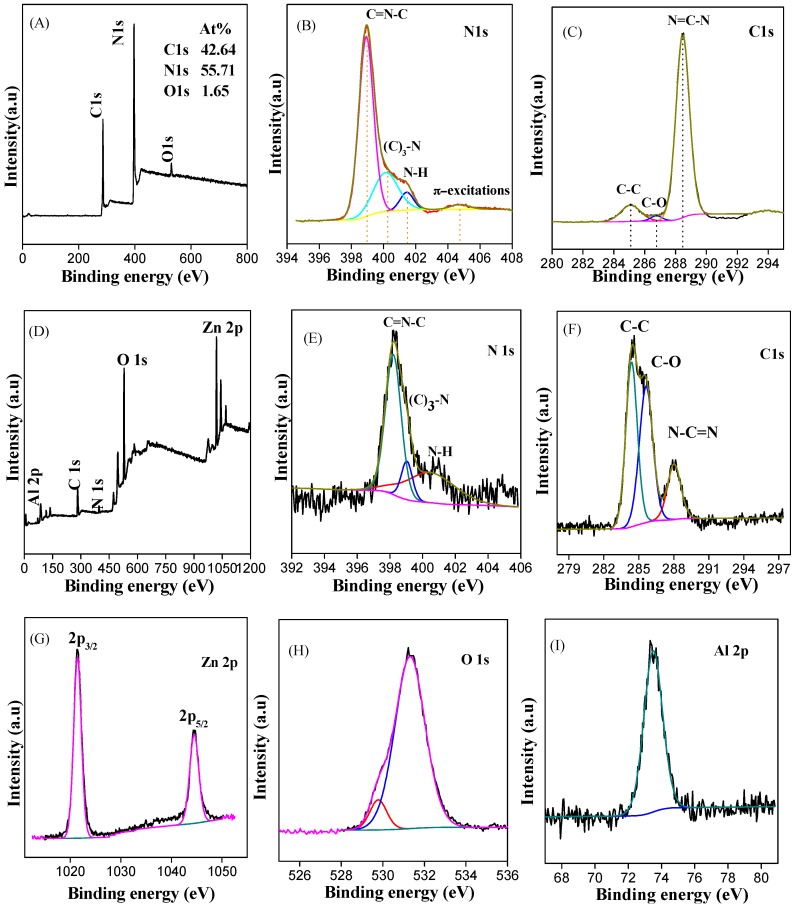
XPS images of the g-C_3_N_4_ and ZnO-LDH@C_3_N_4_ composite. (**A**) Survey spectrum for g-C_3_N_4_; (**B**) High-resolution N 1s XPS spectrum of g-C_3_N_4_; (**C**) High-resolution C 1s XPS spectrum of g-C_3_N_4_; (**D**) Survey spectrum for the ZnO-LDH@C_3_N_4_ composite; (**E**–**I**) are high-resolution XPS spectra for N 1s, C 1s, Zn 2p, O 1s and Al 2p of the ZnO-LDH@C_3_N_4_ composite respectively.

Figure 2A shows the XPS survey spectrum of the g-C_3_N_4_ sample. It can be seen that the g-C_3_N_4_ sample synthesized in this work consisted of mainly C and N elements (the atomic concentration: 42.6% for C and 55.7% for N) with a small amount of oxygen (1.65%). Based on XPS data, the molar ratio of C to N is 0.76, closer to 0.75, suggesting the formula of g-C_3_N_4_. The small amount of oxygen in g-C_3_N_4_ may be caused by incomplete polymerization of urea. Figure 2B shows the peak deconvolution results of N 1s XPS spectrum of g-C_3_N_4_. Three peaks at 399.1, 400.4 and 401.6 eV, attributing to sp^2^-hybridized aromatic N bonded to carbon atoms (C=N–C), the tertiary N bonded to carbon atoms in the form of N–(C)_3_ and N–H side groups, respectively, can be seen [31,32]. A weak peak at 404.5 eV ascribing to the π-excitations is also seen [24]. The high-resolution C 1s XPS spectrum of (Figure 2C) was deconvoluted into three peaks at 288.3, 286.4, and 284.8 eV, corresponding to sp^2^-bonded carbon (N–C=N), C–O and graphitic carbon (C–C), respectively [24,32]. The graphitic carbon C–C at 284.8 eV can usually be observed in carbon nitrides [16,33]. The XPS survey scan of ZnO-LDH@C_3_N_4_ composite is shown in Figure 2D. The presence of O, C, N, Zn and Al in the ZnO-LDH@C_3_N_4_ sample is clearly seen. The atomic concentrations for O/C/N/Zn/Al are 41.59/36.52/4.93/6.67/6.80 (%) from the survey of XPS, which represent the superficial atomic ratios. The high-resolution XPS spectra of N 1s, C 1s, Zn 2p, O 1s, and Al 2p are displayed in Figure 2E–I, respectively. The binding energies for N 1s and C 1s of ZnO-LDH@C_3_N_4_ in Figure 2E,F showed similar deconvolution peaks as pristine g-C_3_N_4_ but with lower energies compared to g-C_3_N_4_, suggesting the strong electrostatic interaction between ZnO-LDH and g-C_3_N_4_ [34,35]. The strong peaks of C–C and C–O were due to the existence of EG and CO_3_^2−^ in ZnO-LDH@C_3_N_4_. The binding energy values of Zn 2p_3/2_ and Zn 2p_5/2_ were fitted with 1022 and 1045 eV in Figure 2G. The high-resolution XPS spectra for O 1s and Al 2p in ZnO-LDH@C_3_N_4_ composites were also fitted in Figure 2H,I respectively. The XPS spectra for ZnO-LDH are illustrated in Figure S1. The binding energy of C 1s in ZnO-LDH only displayed one peak, and no N peaks were detected in ZnO-LDH. Thus, the XPS spectra confirm that layered g-C_3_N_4_ was loaded on ZnO-LDH successfully.

The surface charges of g-C_3_N_4_, ZnO-LDH and ZnO-LDH@C_3_N_4_ were measured by LSE. As listed in Table 1, the g-C_3_N_4_ possessed a positive charge of 20.2 mV attributed to the –NH_2_/NH functional groups at the heptazine rings generated from the incomplete polymerization of g-C_3_N_4_ [36]. The uncondensed amine functional group and the edge cyano-group bring positive-charge characters for g-C_3_N_4_ [37,38]. During the synthetic process with ultrasonic treatment, g-C_3_N_4_ was unfolded and mixed well with NaOH in EG solution, and the pH of the mixture was changed to 13. The zeta potential of the g-C_3_N_4_ was changed into −23.1 mV, attributing to the hydroxyl groups’ adsorption on the g-C_3_N_4_ surface. Therefore, after adding Zn and Al ions, hydroxides generated and anchored onto g-C_3_N_4_. Further solvothermal treatment would reduce the aggregation of the ZnO-LDH@C_3_N_4_ composite. After the hybridization of g-C_3_N_4_ and ZnO-LDH (the weight ratio of g-C_3_N_4_ in ZnO-LDH@C_3_N_4_ is 14.6 wt %, calculated from experimental parameter and confirmed by TG-DTA, see Figure S2), the zeta potential of the composite was shifted to 32.9 mV because of the dominant content of ZnO-LDH (30.4 mV), as illustrated in Figure S3.

**Figure 3:**
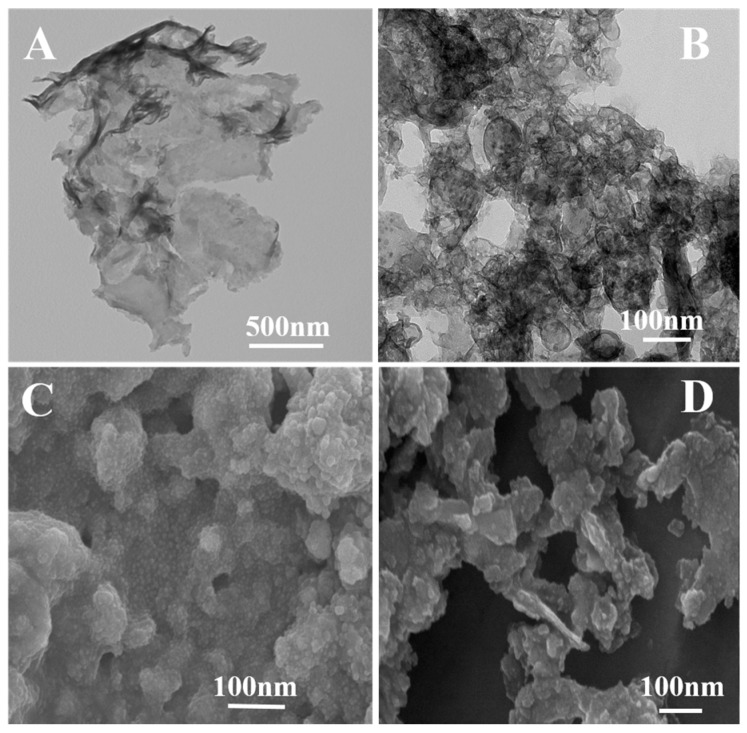
(**A**) TEM image for g-C_3_N_4_; (**B**) TEM image for ZnO-LDH@C_3_N_4_; (**C**) SEM image for ZnO-LDH@C_3_N_4_; (**D**) SEM image for ZnO-LDH.

The morphologies of g-C_3_N_4_, ZnO-LDH, and ZnO-LDH@C_3_N_4_ composite are presented in Figure 3. As shown in Figure 3A, TEM of g-C_3_N_4_ exhibits a flake-like morphology with irregular interstitial pores at the edge of the flake. The porous structure was generated due to releasing NH_3_ and CO_2_ gas during the thermal treatment of urea. The surface area of g-C_3_N_4_ was 128.6 m^2^g^−1^, much higher than the literature reports (Table 1) [36,39]. Such a high surface area of g-C_3_N_4_ might result from the irregular interstitial pore in g-C_3_N_4_. As shown in Figure S4a, bulk g-C_3_N_4_ exhibits overlapped wrinkles and the randomly aggregated g-C_3_N_4_ sheets. (The SEM of bulk g-C_3_N_4_ is given in Figure S4b, which also reveals the layered structure.) After hybridizing ZnO-LDH with C_3_N_4_, TEM image in Figure 3B shows the aggregates of the ZnO-LDH@C_3_N_4_ composite. Different from pristine ZnO-LDH (Figure S4c), ZnO-LDH nanoparticles grew on the g-C_3_N_4_ sheets in ZnO-LDH@C_3_N_4_ composite, suggesting the good affinity between ZnO-LDH and g-C_3_N_4_. Figure 3C,D are the SEM images of the ZnO-LDH@C_3_N_4_ and ZnO-LDH composite, respectively. As seen in Figure 3C, ZnO nanoparticles were evenly distributed on the surface of LDH in ZnO-LDH@C_3_N_4_ composite, which was similar to the characteristic of ZnO-LDH in Figure 3D. Different from ZnO-LDH, a gray veil can be distinguished from the SEM of ZnO-LDH@C_3_N_4_, which represents a covering layer of g-C_3_N_4_ on ZnO-LDH. The average particle sizes of ZnO in ZnO-LDH@C_3_N_4_ and ZnO-LDH are 3 nm and 5 nm respectively, which are coincident with the ZnO crystal sizes (Table 1) calculated from XRD. In the ZnO-LDH@C_3_N_4_ composite, the highly exfoliated g-C_3_N_4_ wrapped up ZnO-LDH particles tightly, which not only provided an intimate contact and cooperation between g-C_3_N_4_ and ZnO-LDH but also increased the surface area of the ZnO-LDH@C_3_N_4_ photocatalyst (152.5 m^2^g^−1^). The HRTEM image and the EDX for the ZnO-LDH@C_3_N_4_ are provided in Figure S5. The lattice spaces for the (100) and (101) planes of ZnO were measured to be 0.282 nm and 0.253 nm, in good agreement with d_(100)_ and d_(101)_, determined from XRD pattern (0.286 nm and 0.252 nm). The EDX analysis shows the presence of Zn, Al, O, C, and N with a Zn/Al atomic ratio of ~1, matching well with the ratio from XPS. The elemental mapping of ZnO-LDH@C_3_N_4_ is presented in Figure S6, exhibiting a uniform distribution of Zn, Al, O, C, and N in the composite. The textural structure of g-C_3_N_4_, ZnO-LDH and ZnO-LDH@C_3_N_4_ studied by N_2_ adsorption-desorption isotherm at 77 K are shown in Figure S7 and listed in Table 1. ZnO-LDH@C_3_N_4_ shows the highest surface area compared to g-C_3_N_4_ and ZnO-LDH, which facilitates the high contact between photocatalyst and dyes. Moreover, the intimate two-dimensional nanojunction between ZnO-LDH and g-C_3_N_4_ favours the photogenerated charge carriers’ transfer between ZnO-LDH and g-C_3_N_4_, which may be a key factor for photocatalytic activities of the ZnO-LDH@C_3_N_4_ photocatalyst.

### 3.2. Adsorption Capacity and Photocatalytic Activity of ZnO-LDH@C_3_N_4_ Composite

#### 3.2.1. Adsorption Performance on Anionic Dye OrgII

**Figure 4:**
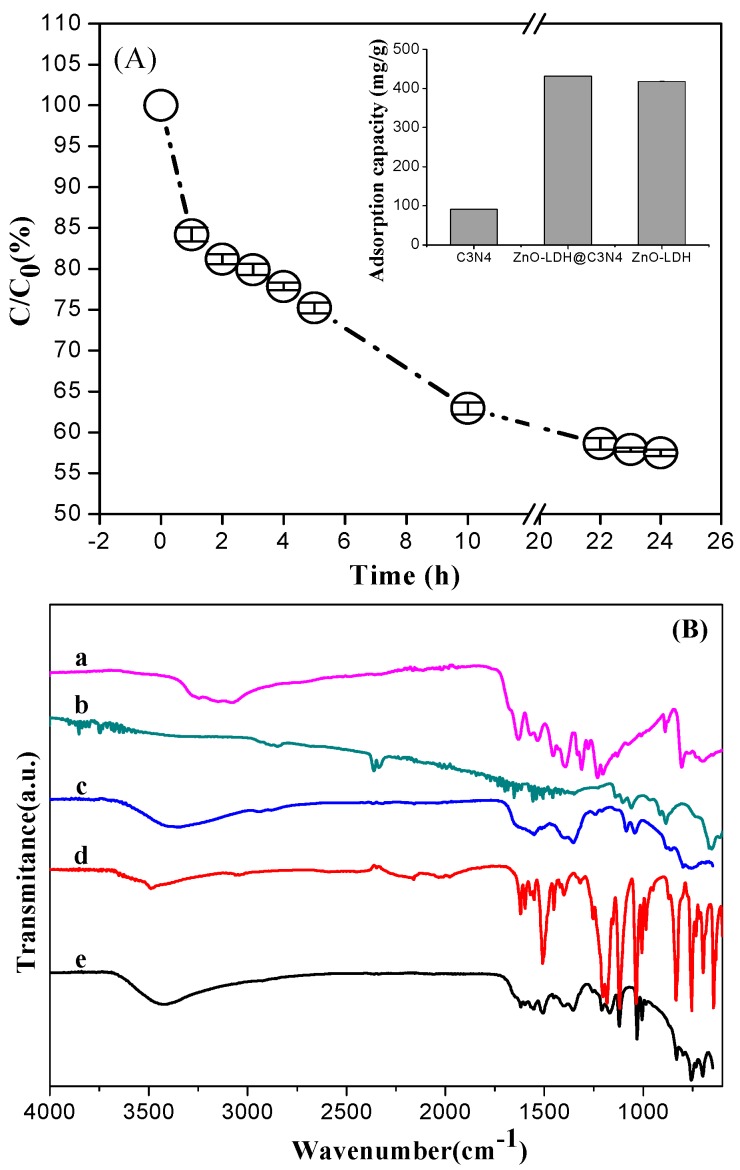
(**A**) The adsorption dynamic of ZnO-LDH@C_3_N_4_ in OrgII adsorption. The insert is adsorption capacity comparison among g-C_3_N_4_, ZnO-LDH@C_3_N_4_ and ZnO-LDH; (**B**) FT-IR spectra of (**a**) g-C_3_N_4_, (**b**) ZnO-LDH, (**c**) ZnO-LDH@C_3_N_4_, (**d**) OrgII, (**e**) ZnO-LDH@C_3_N_4_ after saturated adsorption with OrgII.

Figure 4A exhibits the adsorption dynamics of OrgII on ZnO-LDH@C_3_N_4_. We performed four parallel experiments, so the error bars were also given. The concentration of OrgII firstly reduced from 100% to 85% in 1 h, followed by a moderate decrease in 23% during 9 h, and then slowed down gradually to 55% after 24 h. Thus, the equilibrium contact time was 24 h. Based on the adsorption profile, the adsorption of OrgII on ZnO-LDH@C_3_N_4_ may involve two steps. Firstly, OrgII molecules were quickly adsorbed on the surface of ZnO-LDH@C_3_N_4_ by electrostatic adsorption and π-π conjugation adsorption. Then the massive adsorption of OrgII dyes on ZnO-LDH@C_3_N_4_ happened through anionic exchange between the interlayered EG and OrgII, which gradually slowed down due to the establishment of the charge balance. The adsorption dynamics of OrgII on g-C_3_N_4_ and ZnO-LDH are presented in Figure S8. For g-C_3_N_4_, the absorption equilibrium of OrgII was established in 5 min, indicating the low adsorption of OrgII on g-C_3_N_4_. The adsorption of OrgII on g-C_3_N_4_ was ascribed to the quick π-π conjugation and electrostatic adsorption. ZnO-LDH exhibited a similar adsorption kinetic profile to ZnO-LDH@C_3_N_4_, suggesting that the adsorption of OrgII on ZnO-LDH@C_3_N_4_ was mainly attributed to ZnO-LDH. The inserted graph illustrates the adsorption capacities of ZnO-LDH@C_3_N_4_, g-C_3_N_4_, and ZnO-LDH. ZnO-LDH@C_3_N_4_ showed the highest OrgII adsorption amount with 431.4 mg/g compared to ZnO-LDH and g-C_3_N_4_ with 418.4 and 91.6 mg/g, respectively. This enhancement in adsorption was ascribed to the increased contact between dyes and catalyst derived from π-π conjugation and electrostatic interaction with g-C_3_N_4_ after the combination between g-C_3_N_4_ and ZnO-LDH.

FT-IR can be used to investigate the different situations and types of anionic functional groups between the layers of composites as reported in the literature [31,40]. Therefore, FT-IR was employed to determine the interaction between g-C_3_N_4_ and ZnO-LDH in the ZnO-LDH@C_3_N_4_ composite and its adsorption character. For all the samples, the broad absorption bands located in the range from 3600 to 3000 cm^−1^ are originated from stretching vibration of O-H, C-H, and N-H. These bonds are derived from the hydroxyl group of LDH layers, interlayer EG molecules (O-H and C-H) and uncondensed amino groups in g-C_3_N_4_ [35,41]. The FT-IR spectrum of pristine g-C_3_N_4_ (Figure 4Ba) shows the featured distinctive stretch modes of aromatic CN heterocycles at 1200–1700 cm^−1^ accompanied by the breathing mode of the bending vibration of heptazine rings at 800 cm^−1^, which is corresponding to the reported values [17]. For ZnO-LDH (Figure 4Bb), the characteristic absorption bands of the intercalated molecules like EG are observed at 1500–1800 cm^−1^ and 900–1200 cm^−1^. Furthermore, the band at 650 cm^−1^ can be attributed to the Zn-O-H and Al-O-H stretching vibration in ZnO-LDH. For the composite ZnO-LDH@C_3_N_4_ (Figure 4Bc), a series of peaks observed from 1650 to 1300 cm^−1^ were attributed to the typical stretching modes of CN heterocycles and C=N stretching bonds, which were derived from g-C_3_N_4_ in this composite. It can be clearly seen that the main characteristic peaks for ZnO-LDH and g-C_3_N_4_ appear in ZnO-LDH@C_3_N_4_, suggesting the existence of both ZnO-LDH and g-C_3_N_4_ in the as-prepared composite. The FT-IR spectrum of OrgII (Figure 4Bd) exhibits a sharp peak at 1500 cm^−1^ which can be attributed to the absorption of C=C aromatic stretch. Moreover, several strong absorption bands around 1000–1250 cm^−1^ correspond to –SO_3_^−^ vibrations in OrgII molecules. Furthermore, the bands around 700–850 cm^−1^ are ascribed to the aromatic C-H out-of-plane bending absorption from OrgII. After adsorption of OrgII, the peaks at 810 cm^−1^ assigned to the bending vibration of heptazine rings became sharper and changed into multi-peaks for ZnO-LDH@C_3_N_4_ (Figure 4Be), which could be induced by the molecular cooperation between adsorbed OrgII and g-C_3_N_4_ [24]. The similarly strong absorption bands with OrgII in 1000–1500 cm^−1^ and 700–850 cm^−1^ for ZnO-LDH@C_3_N_4_ (Figure 4Be) indicate that OrgII has successfully intercalated into the LDH layers after adsorption. (The change patterns of FT-IR spectra along with the adsorption time were given in Figure S9.) The successful intercalation of OrgII into LDH was also demonstrated by the shifting XRD pattern in our previous report [6].

#### 3.2.2. Adsorption Performance on Cationic Dye MB

**Figure 5:**
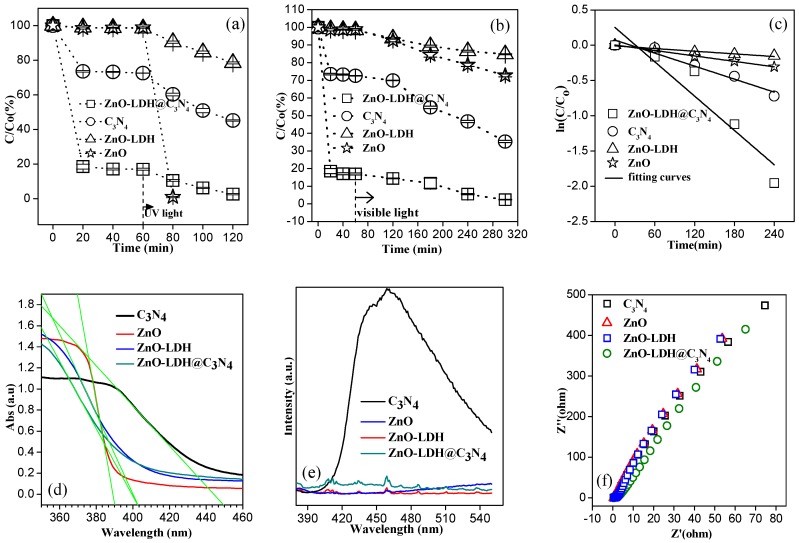
(**a**) Comparison of MB adsorption and photodegradation in water under UV-light over ZnO, ZnO-LDH, g-C_3_N_4_ and ZnO-LDH@C_3_N_4_ respectively; (**b**) Comparison of MB adsorption and photodegradation in water under visible-light over ZnO, ZnO-LDH, g-C_3_N_4_ and ZnO-LDH@C_3_N_4_ respectively; (**c**) Kinetic fit for the degradation of MB with the ZnO, ZnO-LDH, g-C_3_N_4_ and ZnO-LDH@C_3_N_4_ respectively under visible light; (**d**) UV-vis diffuse reflectance spectra of the photocatalysts with corresponding tangent lines; (**e**) Photoluminescence spectra of g-C_3_N_4_, ZnO, ZnO-LDH and ZnO-LDH@C_3_N_4_; (**f**) Electrochemical impedance spectroscopy of g-C_3_N_4_, ZnO, ZnO-LDH and ZnO-LDH@C_3_N_4_ composite.

As shown in Figure 5a, adsorption equilibrium of MB on the ZnO-LDH@C_3_N_4_ composite was established in 20 min, and the adsorption capacity of MB on the ZnO-LDH@C_3_N_4_ composite was 8.0 mg/g, which was much lower than the adsorption capacity for OrgII (431.4 mg/g). The lower adsorption capacity for MB was caused by the positive charges of MB and ZnO-LDH@C_3_N_4_. Different from adsorption of anionic OrgII, MB dye was adsorbed on ZnO-LDH@C_3_N_4_ mainly via the π-π conjugation adsorption instead of electrostatic attraction and ion-exchanged intercalation adsorption. The π-π conjugation adsorption was mainly contributed to the electron-rich properties of g-C_3_N_4_. In addition, the hydrophobicity of C_3_N_4_ may also help MB adsorption on ZnO-LDH@C_3_N_4_. Though the adsorption capacity of MB was low, 80% of MB in solution was absorbed on the ZnO-LDH@C_3_N_4_ composite. Compared with g-C_3_N_4_, ZnO-LDH@C_3_N_4_ composite showed better MB adsorption, ascribing to high surface area of the composite and the uniform g-C_3_N_4_ distribution on ZnO-LDH as well as intimate contact between MB and g-C_3_N_4_. The π-π conjugation adsorption was achieved from the exposure of CN heterocycles on g-C_3_N_4_. Polymeric g-C_3_N_4_ is hydrophobic, which tends to agglomerate in aqueous solution. After hybridizing with ZnO-LDH, g-C_3_N_4_ was unfolded in ZnO-LDH@C_3_N_4_ composite with more exposure of CN heterocycles. The hydrophilic property of ZnO-LDH@C_3_N_4_ promised the intimate contact between catalyst and MB. Therefore, the adsorption amount of MB was superior for ZnO-LDH@C_3_N_4_ composite. In contrast, there was neglected adsorption of MB for ZnO and ZnO-LDH.

### 3.3. Photocatalytic Degradation Performance on MB of ZnO-LDH@C_3_N_4_ under UV and Visible Light

After saturated adsorption of MB, the photocatalytic degradation of MB dyes on commercial ZnO, ZnO-LDH, g-C_3_N_4_, and ZnO-LDH@C_3_N_4_ was undertaken under UV and visible-light irradiation. As shown in Figure 5a, ZnO-LDH@C_3_N_4_ composite removed all of the MB in the water after 1 h UV irradiation. A total of 55.0% of MB was removed by g-C_3_N_4_, and 21.0% of MB was removed by ZnO-LDH. ZnO-LDH@C_3_N_4_ composite showed better photocatalytic performance compared to C_3_N_4_ and ZnO-LDH. However, the commercial ZnO also removed 100% of MB in the water in 20 min under UV irradiation. Interestingly, as shown in Figure 5b, 100% of MB was also removed by the ZnO-LDH@C_3_N_4_ composite in 4 h under visible-light irradiation; on the contrary, only 27.2% of MB could be degraded on commercial ZnO photocatalyst. The low photocatalytic activity of commercial ZnO under the visible light was ascribed to its wide bandgap, which cannot be excited upon visible-light irradiation. The removal rate of MB over ZnO-LDH and g-C_3_N_4_ under visible-light irradiation was 15.3% and 64.1% respectively. The better photocatalytic performance of ZnO-LDH@C_3_N_4_ composite under UV or visible light was ascribed to its narrower bandgap, high surface area and increased contact surface area between MB and the composite. As mentioned in MB adsorption, a large amount of MB was absorbed on the surface of ZnO-LDH@C_3_N_4_; the intimate contact between MB and photocatalyst shortened the mass transfer, which would lead to the dramatic improvement in catalytic performance. Moreover, a combination of ZnO-LDH and g-C_3_N_4_ reduced the bandgap of the composite, which increased the photon utilization efficiency. Most importantly, the heterojunction between ZnO-LDH and g-C_3_N_4_ could facilitate the transfer of excited photoelectrons during the photocatalytic process, which increased the photocatalytic effect fundamentally. Moreover, after the reaction, the color of the sediment changed back to the original yellowish (the color after adsorption of MB was blue), suggesting MB has been fully decomposed.

The photocatalytic degradation kinetics of MB on the photocatalysts were investigated and shown in Figure 5c. The photocatalytic profile of MB on ZnO-LDH@C_3_N_4_ followed pseudo-first-order kinetics plot by the equation:

ln(C/C_0_) = −*k*∙t where *k* is the pseudo-first-order rate constant, C_0_ and C are the MB concentration in solution at times 0 and t, respectively.

After fitting, *k* of commercial ZnO, ZnO-LDH, g-C_3_N_4_ and ZnO-LDH@C_3_N_4_ in Table 1 were 0.0775, 0.0378, 0.185, and 0.487 min^−1^, respectively. The value of *k* gives an indication of the activity of the photocatalyst [42]. ZnO-LDH@C_3_N_4_ had the highest rate constant among all the photocatalysts under visible-light irradiation, almost seven times as high as ZnO. The highest rate constant of ZnO-LDH@C_3_N_4_ further demonstrated the better photocatalytic performance of ZnO-LDH@C_3_N_4_ than that of commercial ZnO. The reusability of ZnO-LDH@C_3_N_4_ was further tested. As shown in Figure S10, the removal rate can still be 90% at the fifth cycle.

UV-vis diffuse reflection spectroscopy (DRS) was used to test the light-harvesting ability of ZnO, g-C_3_N_4_, ZnO-LDH and ZnO-LDH@C_3_N_4_ samples. As shown in Figure 5d, sharp absorption edges for all samples were in the range of 380~450 nm. The bandgap can be inferred from the UV-vis absorption measurements. The bandgaps of ZnO, g-C_3_N_4_, ZnO-LDH and ZnO-LDH@C_3_N_4_ were calculated to be 3.20, 2.72, 3.08 and 3.06 eV, respectively. With the existence of g-C_3_N_4_, we expected that the bandgap of ZnO-LDH@C_3_N_4_ would be tuned to the lower bandgap. However, the bandgap of ZnO-LDH@C_3_N_4_ was 3.06 eV, slightly lower than that of ZnO-LDH (3.08 eV) instead of being close to that of g-C_3_N_4_ (2.72 eV). No change in the bandgap of the ZnO-LDH@C_3_N_4_ composite may be due to the low content of g-C_3_N_4_. The other reason should be that during the solvothermal treatment process, the g-C_3_N_4_ was unfolded and covered on the ZnO-LDH. Therefore, the strong quantum confinement effect (QCE) derived from the highly extended g-C_3_N_4_ increased the bandgap of g-C_3_N_4_ simultaneously [10]. PL spectroscopy was used to investigate the separation efficiency of photoexcited electron-hole pairs [14,25]. All the samples were excited at 360 nm, and the emission spectra were recorded in a range between 380 and 550 nm. As shown in Figure 5e, the g-C_3_N_4_ had the highest emission peak at 470 nm. The emission peak at 470 nm was ascribed to the band-band PL phenomenon with the energy of light approximately equal to the bandgap energy of g-C_3_N_4_. This high intensive emission was attributed to the direct recombination of excitons [43]. Compared to g-C_3_N_4_, the emission intensity of ZnO-LDH@C_3_N_4_ was much lower, suggesting that their e-h^+^ pair recombination rate was much lower. The strong separation of charge carriers resulted in the potentially higher photocatalytic activity for ZnO-LDH@C_3_N_4_. The charge transport process occurs in photocatalyst under dark condition, which directly reflects its capacity to shuttle and convey charge carriers to the targeted reactive sites [25]. Thus, to deeply understand the charge transport behaviour of ZnO-LDH@C_3_N_4_ in the absence of light excitation, EIS measurements were carried out under dark condition. Figure 5f displayed the EIS Nyquist plots of all the samples. As already known, the arc radius on the EIS Nyquist plot reflects the reaction rate on the surface of the electrode. The smaller the arc radius, the more effective the separation of photogenerated electron-hole pairs, and the higher the efficiency of charge immigration across the electrode-electrolyte interface [20,21,44]. Among all the samples, ZnO-LDH@C_3_N_4_ showed the smallest diameter for arc radius, suggesting its lowest resistance for interfacial charge transfer from the electrode to electrolyte molecules. Therefore, EIS measurements were consistent with PL data, demonstrating that the ZnO-LDH@C_3_N_4_ had lower resistance than other samples and made the separation and immigration of photogenerated charges more efficient, indicating the high photocatalytic activity for ZnO-LDH@C_3_N_4_.

### 3.4. Proposed Mechanism under UV and Visible-Light Irradiations

**Figure 6:**
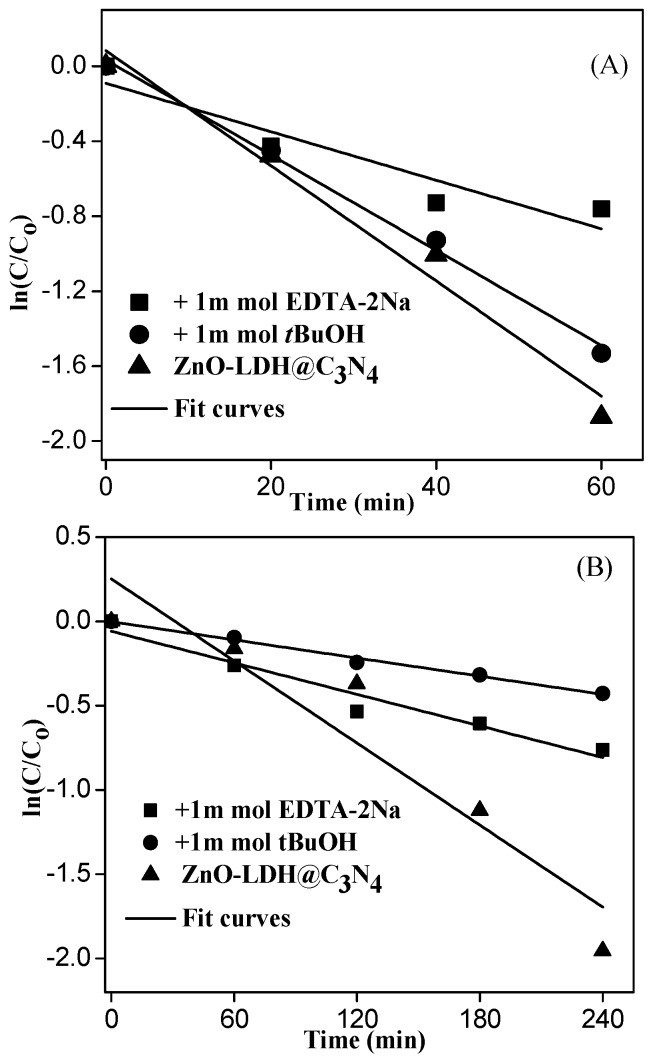
(**A**) The experimental data and the fitting plots of photogenerated carriers trapping in the photodegradation of MB by ZnO-LDH@C_3_N_4_ under UV-light irradiation; (**B**) The experimental data and the fitting plots of photogenerated carriers trapping in the photodegradation of MB by ZnO-LDH@C_3_N_4_ under visible-light irradiation.

As already known, detection of the main oxidative species in the photocatalytic process is a significant tool to reveal the photocatalytic mechanism. The active species generated during the photocatalytic process can be detected through trapping by tertbutyl alcohol (*t*-BuOH) and ethylenediaminetetraacetic acid disodium salt (EDTA-2Na) [23]. *t*-BuOH can be the scavenger for radicals like hydroxyl and superoxide; EDTA-2Na is the scavenger for holes. As shown in Figure 6A, in the ZnO-LDH@C_3_N_4_ system, the MB concentration decreased dramatically upon UV irradiation without adding trapping chemicals. However, the addition of *t*-BuOH only resulted in a small change in the photocatalytic degradation of MB. On the contrary, the photocatalytic activity of ZnO-LDH@C_3_N_4_ was greatly suppressed by the addition of EDTA-2Na. The experiment results indicated that holes were the main oxidative species when the photocatalyst was under UV irradiation. When the photocatalytic reaction was under visible-light irradiation, the situation was reversed. As shown in Figure 6B, the addition of *t*-BuOH suppressed the photocatalytic degradation compared to the addition of scavenger EDTA-2Na. The experiment result suggested that the radicals were the main oxidative species when photocatalytic degradation was processed under visible irradiation.

**Figure 7:**
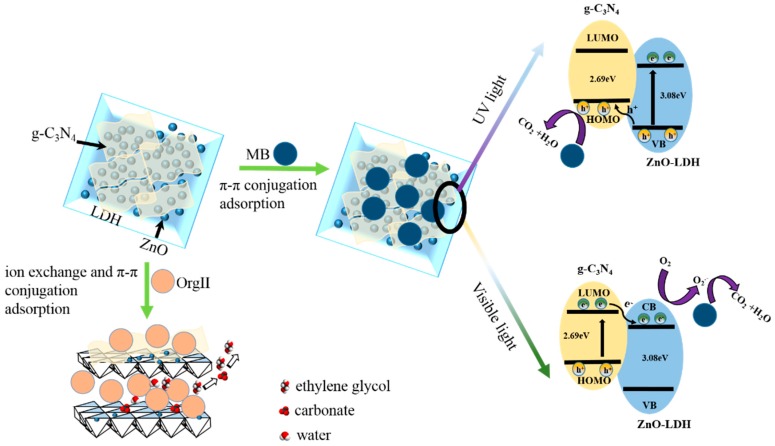
Schematic illustration of the mechanism of uptake of anionic dye OrgII and the charge separation and photocatalytic activity of the ZnO-LDH@C_3_N_4_ under UV- and visible-light irradiation, respectively.

Through the adsorption and photocatalytic performance results, we proposed the following adsorption and photocatalytic mechanism shown in Figure 7. As shown in Figure 7, ZnO-LDH@C_3_N_4_ composites were mixed with OrgII solution and then OrgII was absorbed on the surface of ZnO-LDH@C_3_N_4_ composites via electrostatic interaction and π-π conjugation adsorption and further intercalated into ZnO-LDH via layered adsorption including ion exchange. For cationic MB dye, MB was first adsorbed on the surface of ZnO-LDH@C_3_N_4_ by π-π conjugation. Upon UV irradiation, ZnO-LDH could be excited to produce photogenerated electron-hole pairs. Since the valence band (VB) position of ZnO-LDH is lower than the highest occupied molecular orbital (HOMO) of g-C_3_N_4_, the photogenerated holes on ZnO-LDH could directly transfer to g-C_3_N_4_ [23]. g-C_3_N_4_ is relatively stable with holding holes. The g-C_3_N_4_ with holes would accept electrons from MB degradation and then return to the ground state. Upon visible-light irradiation, g-C_3_N_4_ instead of ZnO-LDH absorbed visible light to induce π-π* transition, transporting the excited-state electrons from HOMO to the lowest unoccupied molecular orbital (LUMO). The LUMO potential of g-C_3_N_4_ is more negative than the conduction band (CB) edge of ZnO-LDH, due to the comparable energy difference between the CB of ZnO-LDH and g-C_3_N_4_; there is a strong thermodynamic driving force for electron transfer from excited g-C_3_N_4_ to ZnO-LDH [45]. The electrons would subsequently transfer to the surface of ZnO-LDH@C_3_N_4_ to react with water and oxygen by generating superoxide and hydroxyl radicals. The radicals can subsequently oxidize the MB into CO_2_ and H_2_O.

## 4. Conclusions

The ZnO-LDH@C_3_N_4_ composite was synthesized via the facile solvothermal method in this work. The introduction of g-C_3_N_4_ on ZnO-LDH significantly improved the adsorption and photocatalytic activities of ZnO-LDH@C_3_N_4_. For OrgII, ZnO-LDH@C_3_N_4_ showed higher adsorption capacity with three synergetic steps including electrostatic and π-π conjugation adsorption followed by ion exchange. Besides, ZnO-LDH@C_3_N_4_ exhibited substantial adsorption of cationic methylene blue (MB) dye and high photocatalytic activities in MB removal under UV and visible light. Moreover, ZnO-LDH@C_3_N_4_ showed stable photocatalytic activities for MB removal in five cycles and 90% of MB was removed at the fifth cycle. The enhanced performance in photocatalytic degradation of MB under UV and visible-light irradiation were induced by the high separation efficiency of photogenerated charges. This work demonstrated that an attractive ternary hybridization of LDH, ZnO and g-C_3_N_4_ could launch new research on a highly active photocatalytic adsorbent for environmental and energetic applications.

## Figures and Tables

**Table 1:** Zeta potential, BET surface area, pore volume, crystallite size and bandgap for the samples and the pseudo-first-order rate constant for MB photocatalytic degradation over different photocatalysts.

Samples	Zeta Potential ^1^ (mV)	S_BET_ (m^2^g^−1^)	Pore Volume (cm^3^g^−1^)	Crystallite Size ^2^ (nm)	Bandgap ^3^ (eV)	*k* ^4^ (min^−1^)
g-C_3_N_4_	40.7	128.6	0.598	-	2.72	0.185
ZnO-LDH	30.4	113.7	0.261	5.2	3.08	0.0378
ZnO-LDH@C_3_N_4_	32.9	152.5	0.298	3.4	3.06	0.487
ZnO	-	-	-	16.1	3.20	0.0775

Note: ^1^ The zeta potential of each sample was measured by testing the suspension with the initial pH value. The initial pH values for g-C_3_N_4_, ZnO-LDH, and ZnO-LDH@C_3_N_4_ aqueous suspensions are 3.29, 7.17 and 6.22 respectively. ^2^ ZnO crystallite size: calculated using Scherrer’s equation: Size = K λ/[FW(s)·cos (θ)]; K is constant = 1 here; λ is the X-ray wavelength = 1.5406 Å. FW(s) is the Full Width at Half Maximum (FWHM) of the sample at θ, here (002) is chosen for the calculation. ^3^ Bandgap of the samples were calculated according to the equation *E*_g_ = 1240/λ. λ is the absorption edge from UV-vis diffuse reflection spectroscopy. ^4^ *k* is the pseudo-first-order rate constant for all the photocatalysts in processing MB photodegradation under visible-light irradiation.

## Supplementary Materials

The following are available online at www.mdpi.com/1996-1944/9/11/927/s1.
