# Supplementary material for: ZnO-Layered Double Hydroxide@Graphitic Carbon Nitride Composite for Consecutive Adsorption and Photodegradation of Dyes under UV and Visible Lights
Source: Materials (Basel). 2016 Nov 15;9(11):927. doi: 10.3390/ma9110927 (PMC5457221; doi:10.3390/ma9110927)
Supplement: Supplementary file 1 [file materials-09-00927-s001.pdf]

Supplementary Materials: ZnO-Layered Double Hydroxide@Graphitic Carbon Nitride Composite for Consecutive Adsorption and Photodegradation of Dyes under UV and Visible Lights

Luhong Zhang, Li Li, Xiaoming Sun, Peng Liu, Dongfang Yang and Xiusong Zhao

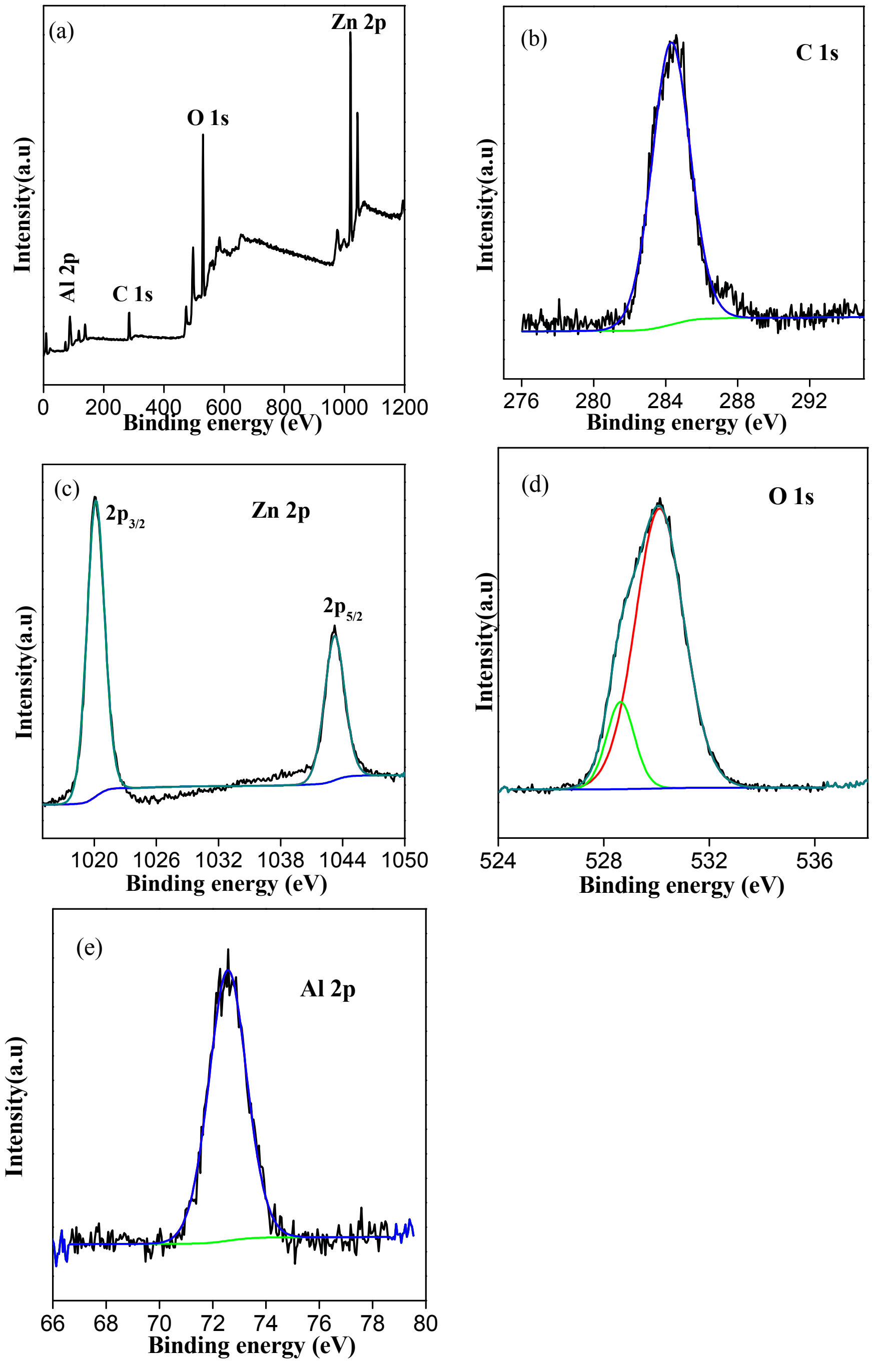

Figure S1. XPS spectra of ZnO-LDH. (**a**) Survey spectrum for ZnO-LDH; (**b**) High-resolution C 1s XPS spectrum of ZnO-LDH; (**c**) High-resolution XPS spectrum for Zn 2p of ZnO-LDH; (**d**) High-resolution XPS spectrum for O 1s of ZnO-LDH; (**e**) High-resolution XPS spectrum for Al 2p of ZnO-LDH.

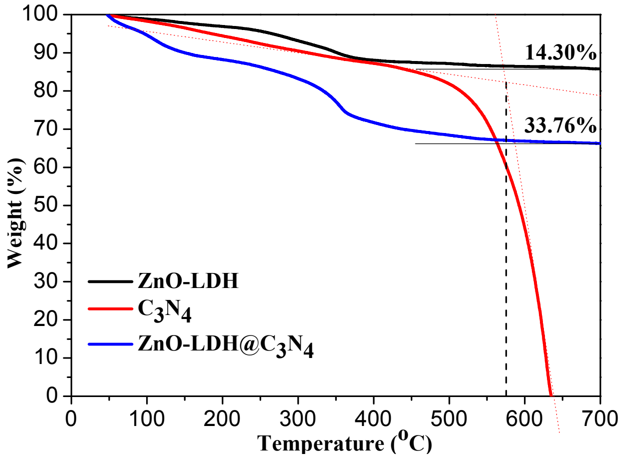

Figure S2. Weight loss of ZnO-LDH, g-C_3N_4 and ZnO-LDH@C_3N_4 composite determined by TGA.

There is a slow weight loss before 500 °C for g-C_3N_4. The first stage with 17.0 wt % mass loss was caused by the loss of surface hydroxyl groups and absorbed water molecules, which was followed by the decomposition of defects and edge functional groups such as uncondensed amine functional group and the edge cyano-group of polymer g-C_3N_4. Then there is a sharp weight loss during 500–600 °C, which indicates the disintegration of the whole g-C_3N_4 polymer. Thus, the crossing point of the two tangents of the two weight loss platforms is supposed to be the weight loss point of g-C_3N_4. The weight loss rate for ZnO-LDH and ZnO-LDH@C_3N_4 are 14.3% and 33.8% respectively. The difference is 19.5%, which is more than the experimental result 14.6 wt % for the content of g-C_3N_4. The larger difference should be ascribed to the larger relative amount of LDH in ZnO-LDH@C_3N_4, which is confirmed by the XRD patterns.

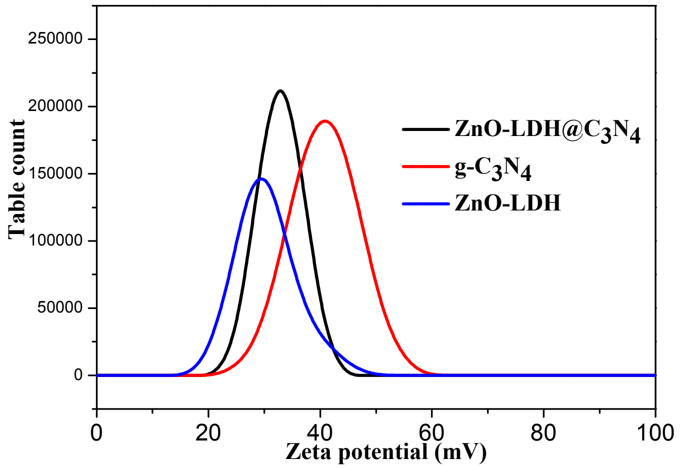

Figure S3. Zeta potential of g-C_3N_4, ZnO-LDH, and ZnO-LDH@C_3N_4 aqueous suspensions.

The zeta potentials of g-C_3N_4, ZnO-LDH and ZnO-LDH@C_3N_4 were measured at room temperature. Typically, each sample powder (20 mg) was dispersed in 20 mL of MQ water by ultrasonication for 30 min. The initial pH values for g-C_3N_4, ZnO-LDH, and ZnO-LDH@C_3N_4 aqueous suspensions are 3.29, 7.17 and 6.22 respectively. When the pH value for g-C_3N_4 aqueous suspension is adjusted into 13.89, the zeta potential is −23.1 mV.

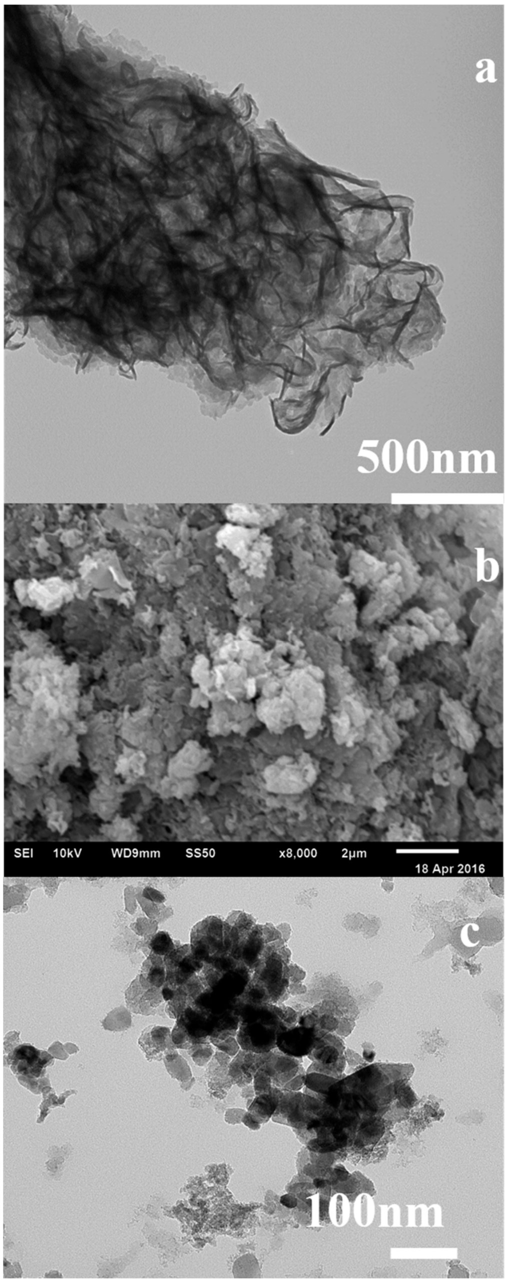

Figure S4. (**a**) TEM image for bulk g-C_3N_4; (**b**) SEM image for g-C_3N_4; (**c**) TEM image for ZnO-LDH.

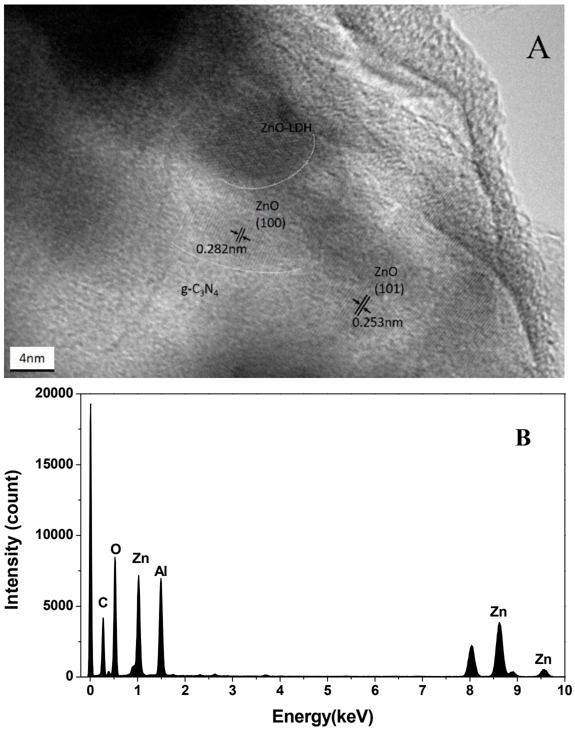

Figure S5. (**A**) HRTEM image for the composite ZnO-LDH@C_3N_4; (**B**) EDX spectrum of ZnO-LDH@C_3N_4.

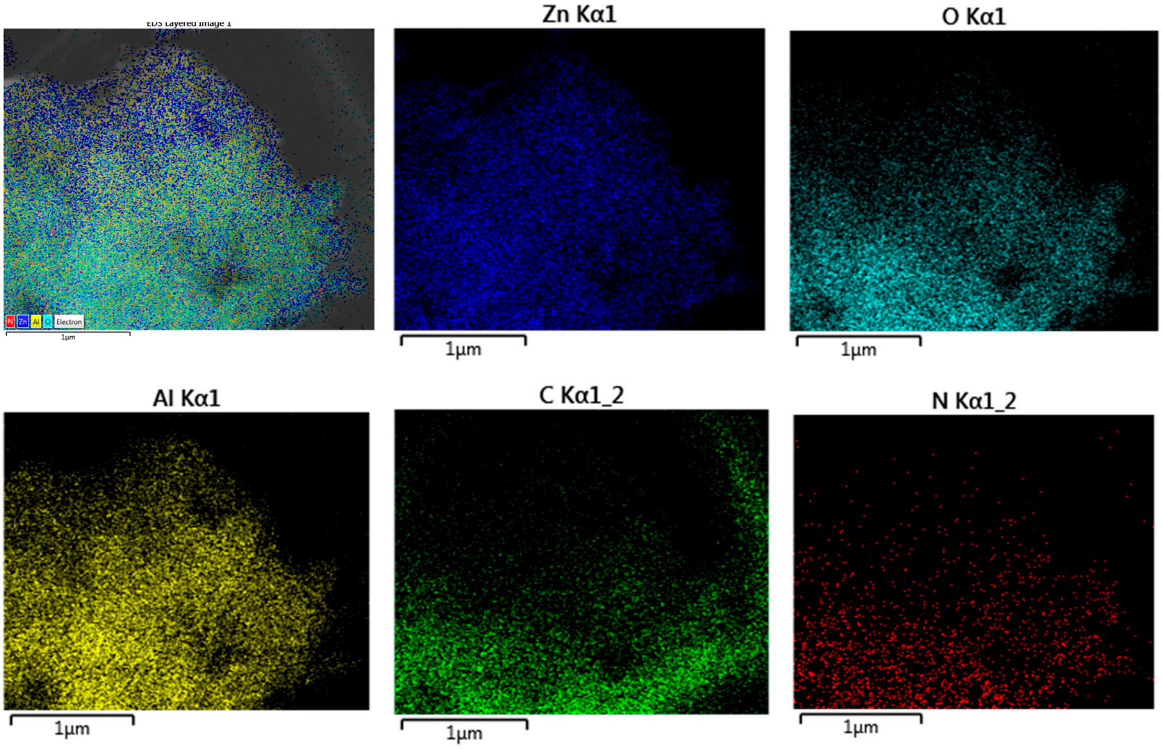

Figure S6. The elemental mapping for ZnO-LDH@C_3N_4.

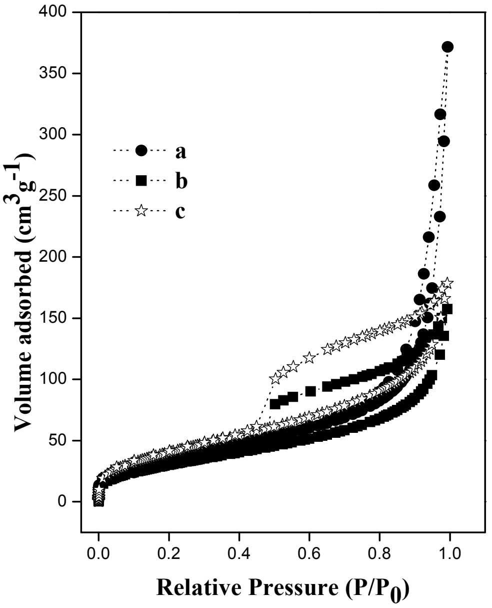

Figure S7. N_2 adsorption/desorption of isotherms of (**a**) g-C_3N_4; (**b**) ZnO-LDH and (**c**) ZnO-LDH@C_3N_4.

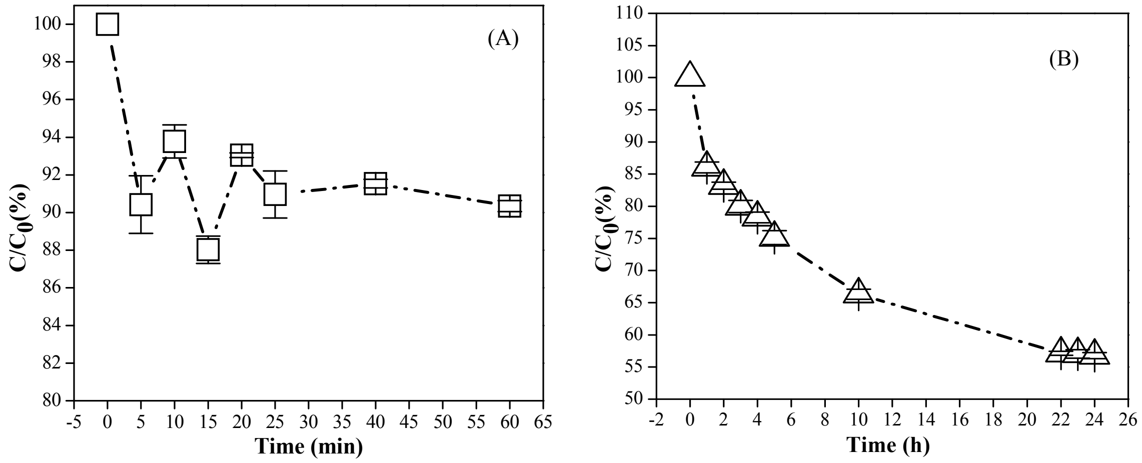

Figure S8. The adsorption dynamics of g-C_3N_4 (**A**) and ZnO-LDH (**B**) in OrgII adsorption.

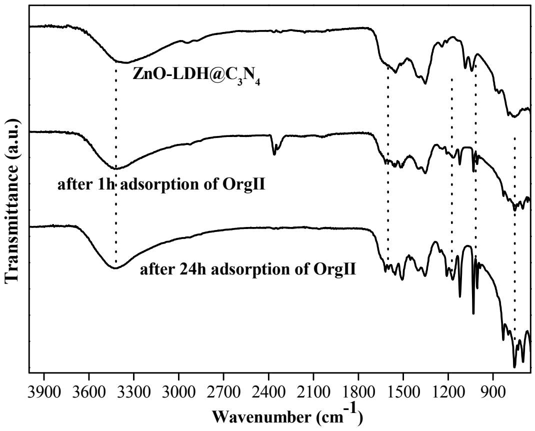

Figure S9. FT-IR spectra of ZnO-LDH@C_3N_4, ZnO-LDH@C_3N_4 after OrgII adsorption in 1 h and ZnO-LDH@C_3N_4 after OrgII adsorption in 24 h.

FT-IR spectra of ZnO-LDH@C_3N_4 samples before and after OrgII adsorption in 1 h and 24 h were shown in Figure S9. The peaks at 810 cm^{-1} assigned to the bending vibration of heptazine rings became sharper along with the adsorption time and changed into multi-peaks after saturated adsorption of OrgII, which may be induced by the molecular cooperation between adsorbed OrgII and g-C_3N_4.

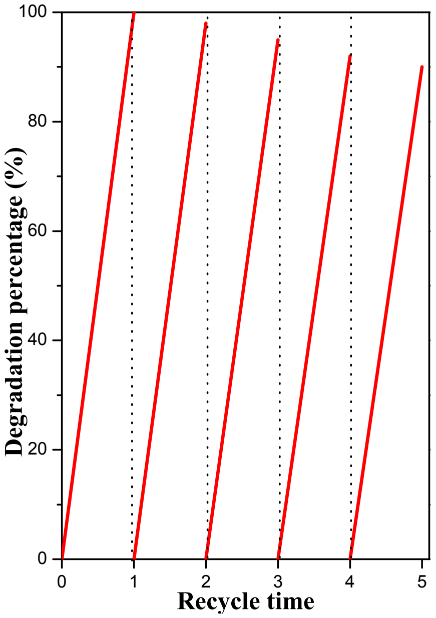

Figure S10. The cycling runs of ZnO-LDH@C_3N_4 in the photodegradation of MB under UV irradiation.
